# Anti-TNF-Alpha-Adalimumab Therapy Had Time Lag of Improvement in Synovial Hypertrophy Compared to Rapid Response in Power Doppler Synovial Vascularity

**DOI:** 10.1155/2017/1658397

**Published:** 2017-09-06

**Authors:** Ying-Chou Chen, Chi-Hua Ko, Jia-Feng Chen, Chung-Yuan Hsu, Wen-Chan Chiu, Tien-Tsai Cheng

**Affiliations:** Department of Rheumatology, Kaohsiung Chang Gung Memorial Hospital, Chang Gung University College of Medicine, Kaohsiung 833, Taiwan

## Abstract

**Objectives:**

The quantification of synovitis is of great significance for follow-up in patients with rheumatoid arthritis (RA). This study aimed to validate the use of power Doppler ultrasonography (PDUS) for evaluating synovial vascularity and synovial hypertrophy for synovitis in patients with rheumatoid arthritis treated with adalimumab.

**Materials and Methods:**

The synovial disease activity and vascularity of RA on both wrists (radio-carpal joint) were assessed using GS and PDUS to derive the composite US scores based on abnormal counts and severity. The relationship between each measure was determined.

**Results:**

The 71 patients who received adalimumab therapy had significantly decreased DAS28, ESR, and CRP. After one month, PD score decreased and then remained low for 12 months. Synovial hypertrophy did not change until 3–6 months after, when it started to improve (*p* = 0.017). By multivariate analysis, sex, age, BMI, and DAS28 did not lead to any difference between synovial hypertrophy and PDUS changes (*p* = 0.498).

**Discussion:**

Composite US markers of synovial hypertrophy correlate significantly to the DAS28 score and ESR/CRP in adult RA. The time needed for synovial hypertrophy to decrease may be up to 3–6 months after adalimumab therapy. Switching to biological therapy before 3–6 months is inappropriate and ineffective.

## 1. Introduction

RA is a clinically and biologically heterogeneous disease. Cellular and molecular heterogeneity exists in the rheumatoid synovium and represents a spectrum of inflammatory and fibroblastic disease processes that span disease course and activity [1]. Some RA patients with active disease may not optimally respond to current anti-inflammatory therapies and may require targeting of different biological pathways for optimal response. It is likely that underlying disease heterogeneity contributes to incomplete drug response and underscores the importance of a deeper understanding of disease pathogenesis to better treat patients.

Adalimumab, unlike traditional disease modifying antirheumatic drugs (DMARDs), has a significant effect on patient disease after one week of treatment. The results suggest that nearly half will respond at 20% level by the second week of therapy, while nearly 90% will respond by the eighth week. However, longer treatment seems to allow patients to achieve higher levels of treatment response [2–4]. In two previous studies, the patients achieved greater disease control (ACR50 response level) in treatment up to 24 weeks. As reported by Keystone et al., this response was maintained over the next 24 weeks [5].

The quantification of synovitis is of great significance for adequate therapy management and follow-up in patients with rheumatoid arthritis (RA). This study aimed to validate the use of power Doppler ultrasonography (PDUS) for evaluating synovial vascularity and synovial hypertrophy for synovitis in patients with rheumatoid arthritis (RA) treated with adalimumab.

## 2. Methods

The Institutional Review Board of Chang Gung Memorial Hospital approved the study protocol, and all participants provided written informed consent. Patients with rheumatoid arthritis (RA) aged 20–70 years and adalimumab therapy approved by the Bureau of National Health Insurance were included. Patients with other systemic illnesses or infections were excluded.

All of the included patients underwent 28 swollen and 28 tender baseline clinical counts. Age, sex, ESR, and CRP level were recorded. The ultrasound scans were scored in random order by an experienced observer without knowledge of the clinical data. Each patient underwent a musculoskeletal systematic multiplanar grayscale (GS) and power Doppler ultrasound (PDUS) examination using a MyLab 70 system (Esaote, Firenze, Italy) equipped with a multifrequency linear array transducer (6–18 MHz). The B-mode frequency was 12–18 MHz for the second and third metacarpal phalangeal (MCP) joint. The power Doppler pulse repetition frequency was 750 Hz with a Doppler frequency of 6.7–11.1 MHz, and low wall filters were used.

At the start of each scanning session at different sites, the focus was positioned at the level of the region of interest. Color gain was adjusted just below the degree that caused the appearance of noise artifacts. The color box was positioned on the level of the assessed site and enlarged to the upper part of the image. The ultrasound assessments were over wrist (radio-lunate joint).

Synovial hypertrophy was graded from 0 to 3 based on the system of Szkudlarek and colleagues [6], with the equivocal “minimal” thickening graded as follows: grade 0, normal; grade 1, synovial thickening bulging over the line linking the tops of the periarticular bones; grade 2, grade 1 plus extension to 1 bone diaphysis; and grade 3, grade 1 plus extension to both bone diaphyses. Synovitis in other joints was graded 0–3 as 0, normal; 1, mild; 2, moderate; and 3, severe, in which grade 1 was defined as synovial thickening in excess of the mean plus 2 standard deviations of normal.

Synovial hyperemia was measured by power Doppler (PD) in each recess, with the maximal score graded according to Szkudlarek et al. as 0, absence; 1, isolated signals; 2, confluent signals in less than half of the synovial area; and 3, confluent signals in more than half of the synovial area. Global ultrasound indices for GS synovitis and PD were calculated by adding the scores from all joints. Ultrasound scans were performed before and at 1, 3, 6, and 12 months after anti-TNF therapy.

### 2.1. Intraobserver Reliability

Intraobserver reliability was evaluated before patient inclusion by scoring for synovitis and PD signal in 20 recorded images of the joints included in the GS and PDUS assessment from 20 patients with active RA.

### 2.2. Statistical Analysis

Wilcoxon test was used to compare the score between baseline and 1 month. Repeated measures analysis of variance (ANOVA) was used to analyze the serial changes in ultrasound score. Multiple linear regression analysis was used to adjust variables to predict radiologic progression.

Intrarater reliabilities were evaluated using a two-way mixed effects model with a consistency definition, in which the between-measure variance was excluded from the denominator variance. Both single and average measure intraclass correlation coefficients (ICC) were calculated for total scores of both GS synovitis and PDUS. In addition, weighted *κ* values were calculated on a joint-by-joint level for both BM and PDUS scores. The ICC and *κ* values were comparable. Scores > 0.60 were considered good and scores > 0.80 were very good.

## 3. Results

From December 2011 to December 2014, 71 patients were approved by the Bureau of National Health Insurance to receive adalimumab therapy. All had severe RA and there was female predominance (78.87%). Their mean age was 54.51 ± 13.16 years, while their mean BMI and the mean DAS28 were 23.32 ± 3.50 kg/m^2^ and 6.52 ± 0.72, respectively (Table 1). All of the patients received methotrexate 7.5–15 mg/week and hydroxychloroquine 400 mg/d. Among them, 19.72% received leflunomide, 9.86% cyclosporin, 15.9% sulfasalazine, and 16.9% azathioprine.

**Figure 1:**
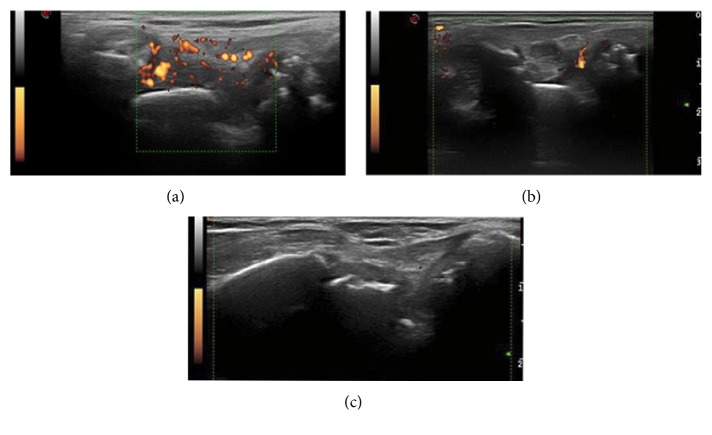
Serial changes after adalimumab therapy at radio-lunate joint (a) baseline (0 month, SH grade 3, SV grade 3), (b) one month (SH grade 3, SV grade 1), and (c) three months after (SH grade 1, SV grade 0). Power Doppler synovitis decreased dramatically on the first month, while gray scale synovitis decreased from the third month.

**Figure 2:**
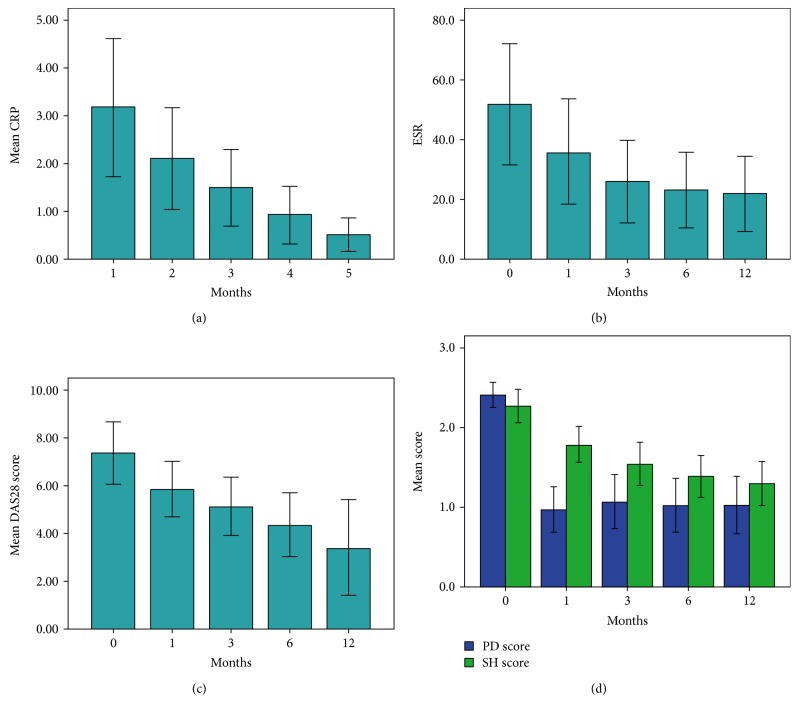
After adalimumab therapy, ESR, CRP, and DAS28 decreased gradually. The PDUS score decreased dramatically within the first month (baseline versus 1 month, *p* = 0.001 by Wilcoxon test) and remained low score (1 month versus 3 months versus 6 months versus 12 months, *p* = 0.379 by repeated measures ANOVA). The GS synovitis decreased less dramatically than PDUS and continued until the 12th month. Using repeated measures ANOVA, the slope was different between the gray scale and power Doppler effect (*p* = 0.008).

After adalimumab therapy, the ESR, CRP, and DAS28 decreased through the following months, while PDUS score decreased dramatically on the first month and remained low score until the 12th month. The synovial hypertrophy decreased less dramatically than PDUS, with the effect slowly from the 3rd to the 6th month (Figures 1 and 2). The PDUS score decreased dramatically within the first month (baseline versus 1 month, *p* = 0.001 by Wilcoxon test) and remained low score (1 month versus 3 months versus 6 months versus 12 months, *p* = 0.379 by repeated measures ANOVA). The GS synovitis decreased less dramatically than PDUS and continued until the 12th month. Using repeated measures ANOVA, the slope was different between the gray scale and power Doppler effect (*p* = 0.008). The trend of improvement in DAS28-ESR/CRP had similar time course with SH. By multivariate analysis, sex, age, BMI, DAS28, methotrexate, hydroxychloroquine, leflunomide, cyclosporin, sulfasalazine, and azathioprine did not lead to any difference between synovial hypertrophy and PDUS changes.

### 3.1. Intraobserver Reliability and Sensitivity to Change of the PDUS Assessments

For synovial hypertrophy and PDUS, the median (range) percentages of intrareader exact agreements were 81.6 and 65.2, respectively, and 89.9 and 79.9, respectively, for close agreements. The weighted *κ* values were median 0.8 for GS synovitis and 0.6 for PDUS.

## 4. Discussion

Adalimumab has a significant effect on patient disease after one week of treatment. Longer treatment seems to allow patients to achieve higher levels of treatment response. In this study, the difference in synovial hypertrophy and PD has some clinical implications. First, synovial hypertrophy has a time lag in improvement compared to PD synovitis and the clinical effect is delayed based on US findings. Thus, when we are starting adalimumab therapy in RA, switching to another biological therapy before 3–6 months is both inappropriate and ineffective.

Second, while synovial vascularity detected by PD is linked to the level of joint inflammation [7, 8], Naredo et al. [9] report a correlation between time integrated values of joint counts for positive synovial vascularity and total joint damage progression at 1 year. The PDUS correlates with vascular endothelial growth factors (VEGF), providing further evidence of a central role for VEGF in synovial neoangiogenesis [10].

The synovial hypertrophy may have different pathogenesis of RA. Ostergaard et al. have demonstrated magnetic resonance imaging- (MRI-) determined synovial membrane volume as a marker of disease activity and a predictor of progressive joint destruction in the wrists of patients with RA [11]. The synovial membrane volume may imply synovial hypertrophy and may have a different mechanism of joint destruction in RA. Shiozawa et al. found under light and electron microscopic studies that the main cells participating in the cartilage destruction at the cartilage-pannus junction were either fibroblast-like or macrophage-like cells [12], Besides, areas of clustered dense staining for fibronectin (Fn) were often observed on the surface of these cells [13]. These findings, in conjunction with the observation of increased levels of this protein in rheumatoid synovial fluids [14, 15], suggest that Fn is secreted by these cells and that the secreted Fn may mediate the attachment of the pannus cells to the collagenous substratum (and its subsequent spreading over the cartilage) as previously observed by Weissmann et al. in a study of neutrophil attachment to a gelatin-coated surface [16]. Grinnell and Feld [17] have shown that cultured fibroblasts secrete Fn onto the surface of substrates, and this secreted Fn interacts with fibroblasts to promote further attachment. Fn has in fact been shown to enhance the attachment and spreading of fibroblasts and macrophages [18] on collagenous substrata. Kurkinen et al. [19] have demonstrated the sequential appearance of collagen followed by Fn in experimentally induced granulation tissue. The sequential appearance of collagen and Fn has also been observed in fibroblast cultures [17]. These findings suggest that collagen-bound Fn may play a role in promoting the assembly of collagen fibrils and in this way may provide an interstitial framework proliferation of inflammatory cells. In the rheumatoid pannus, Fn appears to be present mainly in the active cellular variety of pannus, since preliminary observations of inactive fibrous pannus from 2 additional rheumatoid patients showed only weak staining for this protein. The possibility existed that Fn might mediate the attachment and spreading of the pannus cells over the cartilage matrix in view of the fact that fibronectin was often stained on the cell surfaces of the invading pannus cells. The amount of Fn stained at the C-P junction was decreased in comparison with that stained in the pannus tissue proper. This weak staining at the C-P junction noted by us in the electron microscope has also been observed at this site by Shiozawa et al. [13] using an immunofluorescent staining technique.

One possible explanation for this diminished staining at the C-P junction by the electron microscopic immunoperoxidase method would be that there was limited penetration of the antibodies used for staining at the C-P junction. To avoid this possible source of error, serial ultrathin sections were obtained at depths of less than 15 *μ*m from the cut surface of the specimen, since, as previously shown, there is adequate penetration of HRP antibodies to a depth of at least 30 Am at the level of the C-P junction. Moreover, strong staining was present in other portions of the section which lay at a similar depth from the surface.

In summary, these studies have shown the presence of large amounts of Fn in the interstitial connective tissue of invasive rheumatoid pannus, suggesting a possible role of Fn in promoting pannus proliferation and adherence to cartilage. Strong staining of Fn on the surface of both fibroblast-like and macrophage-like pannus cells suggests that Fn may be produced in situ in the rheumatoid pannus. Fn was also stained at the C-P junction, but the intensity of staining was decreased in comparison with that observed in more superficial areas of the pannus. This decrease may be a result either of enzymatic digestion of Fn at the C-P junction, termination of synthesis as a result of contact of pannus cells with cartilage matrix, or transfer of Fn from pannus cell surfaces to cartilage matrix collagen following contact between the cell and the cartilage.

In a study, adalimumab cannot inhibit fibronectin [20], so we speculate that this reason for synovial hypertrophy was related to fibroblast and macrophage and showed a time lag of synovial hypertrophy to achieve improvement than PDUS score. Besides, in our previous study [21], we showed that no improvement in synovial hypertrophy at one month after adalimumab therapy can predict future joint damage, so synovial membrane volume plays an important role in this pathogenesis of bone destruction.

Adherence to adalimumab had important value. First, the erosive progression is arrested. Dohn et al. observe no statistically significant change in ultrasonography erosion scores at 6 or 12 months. The majority of patients have negative or unchanged erosion scores, strongly suggesting that the erosive progression is arrested [22].

Second, the OPTIMA trial treatment to a stable low disease activity target results in improved clinical, functional with adalimumab therapy [23].

Keystone et al. report a 10-year study where treatment initialization with adalimumab significantly limits radiographic progression, joint erosion, and joint space narrowing [24]. Initiation prior to the development of RA treat-to-target recommendations, the 10-year PREMIER study reinforces adherence to current recommendations for treatment of patients with early, aggressive RA.

Using ultrasound Doppler measurements that predict success of treatment with anti-TNF-alpha has been a trend in monitoring biological therapy in patients with RA [25–27]. Ultrasound color Doppler is associated with synovial pathology in biopsies from hand joints in RA patients [28]. Kawashiri et al. have observed strong correlations between the grades of US-proven articular synovitis and MRI-proven osteitis score in patients with RA, between the presence of US-proven bone erosion and MRI-proven osteitis score in patients with RA, and joint injury assessed by US correlates with MRI proven osteitis in patients with RA [29]. For Fukae et al., changes in local synovial vascularity (SV) have prognostic value for local joint destruction that may lead to meticulous control of inflammation. The evaluation of SV provides important information and contributes to the clinical practice of managing RA [30, 31].

So under the treat-to-target recommendations, the use of validated composite measures of disease activity, which include joint assessments, is needed in routine clinical practice to guide treatment decisions; ultrasound is one of the modalities for assistance in joint assessment. However, treat-to-target recommendations 8, until the desired treatment target is reached, drug therapy should be adjusted at least every three months. Our findings under ultrasound guide implicate that adjusted period more than 6 months may be a more reasonable time, at least in adalimumab use.

In conclusion, composite US markers of synovial hypertrophy correlate to the DAS28 score and ESR/CRP in adult RA, but the time to decrease synovial hypertrophy may be delayed by three months after adalimumab therapy. When starting adalimumab therapy in RA, switching to other biological therapy before 3–6 months is not recommended.

## Figures and Tables

**Table 1:** Baseline demographic and clinical characteristics of patients with rheumatoid arthritis who received anti-TNF-alpha-adalimumab therapy.

Characteristic	Number	%	Mean ± SD
Age (yrs)			54.51 ± 13.16
Body mass index (kg/m^2^)			23.32 ± 3.50
DAS28 score			6.52 ± 0.72
Sex			
Male	15	21	
Female	56	79	
Smoking			
No	67	95	
Yes	4	5.2	
Alcohol consumption			
No	68	96	
Yes	3	4.2	
*Use of other RA mediations*			
Methotrexate			
No	0	0	
Yes	71	100	
Hydroxychloroquine			
No	0	0	
Yes	71	100	
Leflunomide			
No	57	80	
Yes	14	20	
Cyclosporin			
No	64	90	
Yes	7	9.9	
Sulfasalazine			
No	59	84	
Yes	12	17	
Azathioprine			
No	59	83	
Yes	12	17	

RA: rheumatoid arthritis; DAS28: disease activity score in 28 joints.
